# The impact of prison deinstitutionalization on community treatment services

**DOI:** 10.1186/s40352-015-0021-7

**Published:** 2015-05-06

**Authors:** Beverly D Frazier, Hung-En Sung, Lior Gideon, Karla S Alfaro

**Affiliations:** 1 Department of Law, Police Science & Criminal Justice Administration, John Jay College of Criminal Justice, 524 West 59th Street, New York, NY 10019 USA; 2 Department of Criminal Justice, John Jay College of Criminal Justice, 524 West 59th Street, New York, NY 10019 USA

**Keywords:** Reentry, Treatment services, Deinstitutionalization, Recidivism

## Abstract

**Background:**

With one in every 108 Americans behind bars, the deinstitutionalization of prisons is a pressing issue for all those facing the daunting challenges of successfully reintegrating ex-offenders into both their communities and the larger society. Given the strong evidence that treatment services, such as mental/behavioral health, alcohol/substance abuse, and primary healthcare may reduce recidivism, the large number of prisoner releases highlights the need for adequate treatment services in the community. It is within this context that the current study aims to examine the effects of prison deinstitutionalization on community based intervention modalities.

**Methods:**

This study set out to address a set of fundamental research questions in the current climate of reversing the 40-year upward trend in prison population. This thread of inquiry is based on a hydraulic model of institutionalization of transinstitutionalization. This hydraulic framework posits that there are many overlaps between public safety and mental health needs, and that psychiatric institutionalization and penal institutionalization are functionally dependent. Longitudinal data with annual standardized measures such as rates and percentages for this change modeling were obtained from a number of national data programs for all 50 states. Our analytical focus concentrated on the second half of the decade of the 2000s.

**Results:**

Change in the state imprisonment rate was negatively correlated with change in the rate of substance abuse treatment admissions (r = -0.24; p < .05) and the change in the rate of inpatient admissions in state psychiatric hospitals (r = 0.10; p > .05) as predicted. However, only the bivariate association between imprisonment and substance abuse treatment admissions attained the conventional threshold of statistical significance. Holding constant the direct and indirect effects of changes in the rates of violent crime and illicit drug use, change in prison population was negatively associated with changes in the rate of substance abuse (unstandardized coefficient = -0.891; p < 0.05) and mental health admissions (unstandardized coefficient = -0.509; p > 0.05) in the community.

**Conclusion:**

By using a path analysis of the hydraulic model, we argue that social systems, similar to water moving in closed tubes, aspire to equilibrate. In other words, a decrease in prison population will not go without a corresponding increase in community mental health and substance abuse services. Social voids like those created by deinstitutionalization must be filled; and with states deinstitutionalizing offenders the toll is on their corresponding communities to address the needs of those offenders who are reentering after being incarcerated. In devising a policy and practice strategy to address the projected increase in the reentry population, leadership within communities for social and supportive services to ex-prisoners, specifically treatment services should be of primary concern.

## Background

With one in every 108 Americans behind bars (Glaze and Herberman 2013), the deinstitutionalization of prisons is a pressing issue for all those facing the daunting challenges of successfully reintegrating ex-offenders into both their communities and the larger society. Of the 2.2 million persons incarcerated, when considering race, age and gender, the disparity is staggering for young minority males (Glaze and Herberman 2013). In 2012, Black men were six times more likely and Hispanic men were 2.5 times more likely to be imprisoned than white males (Carson and Golinelli 2013). For the more than 700,000 individuals released annually, who are returning to communities in metropolitan areas with high concentrations of ex-offenders, the challenges may be overwhelmingly greater than for the larger reentry population. Many of these communities are facing high levels of socioeconomic distress and are dealing with already strained resources (Clear 2007). Complicating the reentry process and increasing the threat of unsuccessful reentry even more, present deinstitutionalization trends indicate an increase in the reentry population, in the not so distant future.

Given the increase in state and municipal budget constraints (Archibold 2010; Riccardi 2009; Warren 2008) and the repeal of certain draconian drug laws, such as the Rockefeller Drug Laws, it is likely that offenders’ sentences may be shorter than in previous years (Fields 2009; Kendrick 2011; Peters 2009) (see Fair Sentencing Act of 2010, National Criminal Justice Commission Act of 2011). The result could well be an increase in the number of ex-offenders released over the next few years, thus increasing the count and density of ex-offenders in many communities across the nation, making successful reintegration an even greater strain on community resources.

The United States is already experiencing an increase in ex-offenders returning to communities nationwide, as documented in recent Bureau of Justice Statistics reports on the prison population (Glaze 2011). The combined U.S. state and federal prison population, numbered 1,612,395, decreased 0.3 percent in 2010, which was the first decline of prison population experienced since 1972 (Guerino et al. 2011). After sharp unprecedented increases in the 1980s and 1990s, the incarceration rate has since grown at a slower pace, and is in its fourth year of decline from 2009–2012 (Glaze and Herberman 2013). Over the same period, prison release increased by about the same percentage (2.2%); thereby, slightly increasing the rate of those returning from prisons and jails back to their communities. Previously, in 2008, the number of ex-prisoners returning to the community reached an all-time high (Lattimore et al. 2010).

Reports indicate that out of the individuals who serve a prison term, 90–95 percent will return to the community at least one time – most of whom will be rearrested within three years and returned to prison for new crimes or parole violations (Bureau of Justice Statistics 2007). As prisoners are released from incarceration, the community is often called upon to provide treatment and other services in order to reduce recidivism. These tasks have become increasingly overwhelming because there are more than seven million adults in the United States under some form of criminal justice supervision (Maruschak 2012).

Data shows that many offenders currently under criminal justice supervision, either incarcerated or supervised in the community, are diagnosed with multiple health issues, including mental health (Fazel and Danesh 2002), substance/alcohol abuse (Welsh 2011; Sung et al. 2013), and deteriorating physical health (Dretsch 2013; Freudenberg 2001; Watson et al. 2004). Given the strong evidence that treatment services, such as mental/behavioral health, alcohol/substance abuse, and primary healthcare may reduce recidivism, the large number of prisoner releases highlights the need for adequate treatment services in the community. The importance of these treatment services lies in their relationship to recidivism. Recidivism is a vital measure because it represents a significant societal cost, and it is a known empirical observation that, while it might not be true in some communities, nationally, most released prisoners will re-offend. In addition, the time it takes for someone to recidivate for a drug-involved offense is much shorter than other types of offenses (Holleran 2002). This observation suggests that addiction counseling may be exceptionally critical to reducing recidivism rates. However, many states are not able to meet the multiple needs of those returning from prison and jail due mainly to the lack of proper resources, specifically for treatment services (Frazier 2008). This is particularly true for those communities disproportionately impacted by high counts and densities of returning ex-offenders (Gideon 2010).

## Literature Review

### Overwhelming need for treatment services

While released prisoners have many types of needs (e.g. education, employment, housing, treatment services, etc.), addiction counseling for alcohol and substance abuse is considered one of the most prominent. In fact, sixty to eighty percent of incarcerated offenders have been involved with drug use and, of this, approximately only 11 percent have received any type of professional treatment since admission (Center on Addiction and Substance Abuse 2010). Studies have shown that approximately half of all prisoners are diagnosed with some form of substance abuse (Chandler et al. 2009). Based on two observations made by Spjeldnes et al. (2012), it is reasonable to assume that at least some of these individuals will still need treatment once they are released from prison. Firstly, nearly three-quarters of state prisoners, who are expected to be released within the next year, reported a history of drug and/or alcohol abuse. Secondly, only one in six inmates received treatment for alcohol/substance abuse or mental/behavioral problems and even fewer received treatment after release (Spjeldnes, et al. 2012). In prison, the mental and physical problems of inmates go undiagnosed unless the inmate complains, and even then, they may not receive treatment for reasons such as the lack of adequate services (Ross et al. 2011). Since ex-offenders report substance problems in prison but do not receive treatment, the conclusion that they may need treatment in the community upon release is supported.

Unfortunately, many released prisoners do not receive treatment upon release. In a Maryland study, Visher et al. (2004) demonstrated gradually decreasing enrollment of prisoners in drug or alcohol treatment programs. Twenty-seven percent of the approximately 300 surveyed respondents participated in such programs before release. After 30–45 days post-release, the percent dropped to 18 percent, with a further drop to eight percent after four to six months following release (Visher, et al. 2004). Similarly, 12.1 percent of the surveyed respondents indicated having had some type of outpatient substance abuse treatment in the past 30 days, and only three percent had such treatment four to six months after release. It is clear that treatment services may be useful for released prisoners because 63 percent of subjects in the study reported using alcohol and drugs more often or in larger amounts than they intended after being released (Visher, et al. 2004).

In addition to addiction treatment, released prisoners may require mental health treatment. Research based on data from personal interviews estimates that more than half of all inmates (including 56 percent of state prisoners, 45 percent of federal prisoners and 64 percent of jail inmates) have a mental health problem (Hawkins et al. 2010). Furthermore, statistics show that 14 percent to 31 percent of the nine million inmates released from jail every year have a diagnosis of mental illness (Draine et al. 2010). These individuals with mental illnesses may strain the capacity of community mental health systems upon release because of their sheer numbers. This strain is complicated by the observation that offenders with mental health issues often have trouble complying with advice from mental health professionals. Using a sample consisting of 301 offenders from Allegheny County Jail in Pennsylvania, who were 30 days from release, Spjeldnes, et al. (2012) tracked the men after their release in an effort to determine factors that could predict recidivism. The study examined personal characteristics of the sample and found that 92 percent of inmates, who had been diagnosed as severely mentally ill from the sample prison population, were known to be non-adherent to psychiatric medications before their current arrest and 95 percent had a prior arrest. It is possible that this non-adherence is due to inadequate supervision from mental health professionals and deficient mental health care system within prison facilities. Some estimates suggest that up to 11 percent of prisoners may suffer from dual diagnoses or co-occurring diseases – substance abuse and mental illness being the most prevalent (Edens et al. 1998). Clearly, these observations suggest that there might be resources to support only one of the illnesses, and that released prisoners receive worse or no treatment relative to the general population.

Furthermore, there is also evidence that the spread of HIV in the community may be disproportionately tied back to released prisoners. For example, 39 percent of all HIV-positive women in Rhode Island were first diagnosed while incarcerated, which suggests that establishing a link between prison and community follow-up treatment is essential (Flanigan et al. 1996). As described, the treatment needs for released prisoners are dire and diverse. Ex-prisoners leave a fully controlled environment in prison and return to one in which they rely on the community to help them in the reintegration process. If needs are not met, returning prisoners may return to crime, and the revolving door of the criminal justice system begins to turn (Morani et al. 2011). The community’s inability to provide adequate services to released prisoners is further challenged by the deinstitutionalization of persons with mental illness that began in the 1960s.

### Deinstitutionalization in context

Deinstitutionalization has three parts, which includes “the release of individuals” from the institution into the community, “their diversion” from entering the institution, and “the development of alternative community services” (Lamb and Bachrach 2001, p. 1039). The key to successful deinstitutionalization and reentry is proper planning and implementation in all stages of reentry, which sometimes occur simultaneously. Mental health deinstitutionalization was driven by both a desire to maximize effectiveness of resources and a hope to make the process of treating the mentally ill more humane. The passage of the "Community Mental Health Act of 1963 was based on the optimism that former residents of state psychiatric institutions could find meaningful restoration of their lives back in the community, with the help of local mental health clinics" (Bond et al. 2004, p. 572). Unfortunately, the act did not work as intended because of poor implementation of community services (Bond et al. 2004). Thus, the community may still be suffering from its inability to properly treat mental health after the deinstitutionalization in the 1960s. This is exceptionally important in the context of released prisoners because, as stated earlier, the prevalence of mentally ill offenders incarcerated in the nation’s jails and prisons present an enormous challenge on communities in which these individuals will be released to (LaVigne and Mamalian 2004). One may argue that such a challenge is an indication of the deinstitutionalization pendulum.

**Figure 1:**
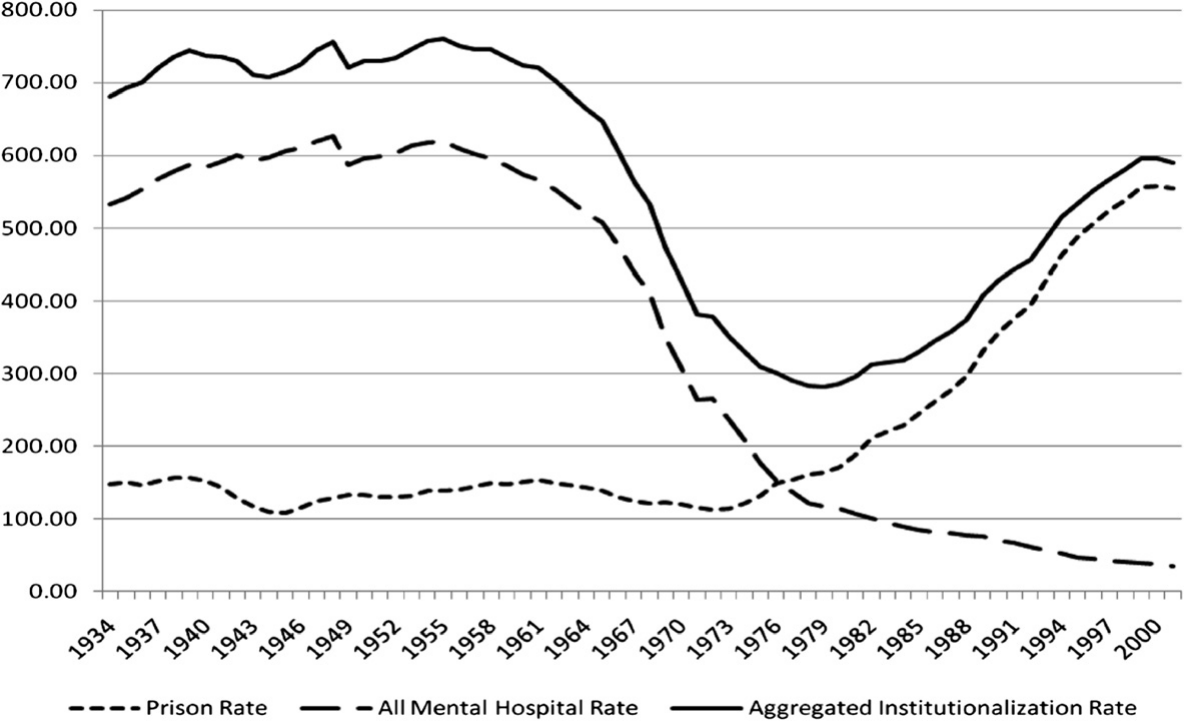
**Rates of institutionalization in the United States (per 100,000 adults), 1934–2001.** (Reprinting by permission of author, Bernard E. Harcourt).

As a result of this deinstitutionalization movement, more mentally ill individuals, who might have previously been treated in mental institutions, were being sent to prison, and, unfortunately, prison often worsens mental illness before individuals are released back into the community as seen in Figure 1 (Nicholas and Bryant 2013; Harcourt 2011). A common theme in both mental health and prison deinstitutionalization is that the community is often unprepared to meet the needs of these reentry populations. This is significant because institutions often can divert released patients or prisoners from re-institutionalization by connecting them to community resources, but the community still has not learned how to adjust to treatment needs of these individuals. In the case of the mentally ill, it appeared that community institutions did not have the skills necessary to treat the mentally ill (Turner and Tenhoor 1978).

It is within this context that the current study aims to examine the effects of prison deinstitutionalization on community based intervention modalities. As the deinstitutionalization of mental institutions in the 1960s is believed to have inversely impacted incarceration, it is hypothesized that the same relationship exists – that is, that there is an inverse relationship between prison deinstitutionalization and the demand for treatment services in the community.

## Methods

### Research questions and hypotheses

This study set out to address a set of fundamental research questions in the current climate of reversing the 40-year upward trend in prison population. How does the incipient reduction in prison population in different states affect mental health and substance abuse treatment needs in local communities? How does the deinstitutionalization of criminal offenders create new demands for substance abuse and mental health services in our communities? This thread of inquiry is based on a hydraulic model of institutionalization of transinstitutionalization.

This hydraulic framework posits that there are many overlaps between public safety and mental health needs, and that psychiatric institutionalization and penal institutionalization are functionally dependent: squeezing one system of institutionalization can produce a bulge in another system of institutionalization (Prins 2011). Just as earlier deinstitutionalization, which when coupled with inadequate funding of community-based services for individuals in need of psychiatric treatment led to the criminalization of mental illness and attendant increases in prison population (Earley 2006), current deinstitutionalization of criminal offenders may lead to the re-institutionalization of psychiatric patients if insufficient financial and political resources are committed to the support of offenders with serious mental health or drug abuse problems in the community. The involvement of individuals with chronic and severe mental health or substance abuse problems in criminal justice processes is often known as “entrenchment” because they remain imprisoned longer, are less likely to receive community sanctions, and are much more likely to violate their probation or parole conditions and return to jail or prison than other offenders charged with comparable offenses (Prins and Draper 2009). It is usually assumed that the size of this vulnerable population at the intersection of public health care and criminal justice supervision has been relatively stable over time. Their institutional destination is mostly determined by the ideological climate and policy environment of the time.

Two hypotheses are thus formulated to explain this hydraulic linkage between prison population downsizing and mental health/substance abuse service needs across the 50 states of the union: (1) Change in the rate of state imprisonment is negatively associated with change in the rate of substance abuse treatment admissions in local communities; the greater the decrease in the size of the prison population, the greater the increase in the relative number of substance abuse treatment admissions in the state, and vice versa; and (2) change in the rate of state imprisonment is negatively associated with change in the rate of mental health treatment admissions in local communities; the greater the decrease in the size of the prison population, the greater the increase in the relative number of mental health treatment admissions in the state, and vice versa. These hypotheses were tested with a national data set covering years 2005–2010.

### Data

Longitudinal data with annual standardized measures such as rates and percentages for this change modeling were obtained from a number of national data programs for all 50 states. Change was operationalized as the difference between two measurements in time. Since efforts at reversing the long trend of growing prison population started to gain momentum in the middle of the first decade of the 21^st^ century (Austin 2010; The Pew Center on the States 2010), our analytical focus concentrated on the second half of the decade of the 2000s.

The two exogenous variables, change in the rate of violent crime between 2005 and 2010 and change in the past month prevalence of illicit drugs between 2005 and 2010, were constructed from annual standardized measures of arrests for violent crime and self-report past month illicit drug use from the Uniform Crime Reports (UCR) of the Federal Bureau of Investigation (FBI); and the National Survey on Drug Use and Health (NSDUH) of the Substance Abuse and Mental Health Services Administration (SAMHSA) respectively (FBI 2006, 2011; SAMHSA 2008, 2012a). Change in the rate of state imprisonment between 2005 and 2010, the mediating variable in the path model, was computed based on the annual rate of incarceration in state prisons reported in the National Prisoner Statistics (NPS) by the Bureau of Justice Statistics (BJS) (Harrison and Beck 2006; Guerino, et al. 2011). A two-year time lag was built into the construction of the last two endogenous variables to specify the temporal order in the sequencing of events. First, change in the rate of substance abuse treatment admissions in the community between 2007 and 2010 was computed from SAMHSA’s Treatment Episode Data Set (TEDS) (SAMHSA 2012b). Finally, change in the rate of in-patient admissions in state psychiatric hospitals between 2007 and 2010 was calculated from the Mental Health National Outcome Measures (NOMS) by the Community Mental Health Services (CMHS) Uniform Reporting System (URS) of SAMHSA.

Table 1 provides a descriptive summary of the variables used in this analysis. The change in state imprisonment rates across the country averaged 0.72 percent between 2006 and 2010, whereas rates of statewide substance abuse admissions and inpatient psychiatric admissions changed by −5.59 percent and 9.41 percent respectively (see Table 1). These fluctuations in institutional statistics took place in a wider context of changes in violent crime and substance abuse rates in the general population between 2005 and 2010, which were −4.02 percent and 4.55 percent respectively.

**Table 1:** **Description of variables (N = 50 states)**

Variable name	Description	Mean	Std. Dev.
*Endogenous Variables*			
Imprisonment Rate	Percent change in the rate of state imprisonment per 100,000 population between 2006 and 2010 (Bureau of Justice Statistics)	0.72	8.52
Substance Abuse Admissions	Percent change in the rate of statewide substance abuse treatment admissions per 100,000 population between 2007 and 2010 (Treatment Episodes Data Set - Substance Abuse and Mental Health Services Administration)	−5.59	29.51
Mental Health Admissions	Percent change in the rate of inpatient admissions in state psychiatric hospitals per 1,000 population between 2007 and 2010 (Community Mental Health Services – SAMHSA)	9.41	43.61
*Exogenous Variables*			
Violent Crime	Percent change in the rate of violent offenses per 100,000 population between 2005 and 2010 (BJS)	−4.02	23.63
Illicit Drug Use	Percent change in the past month prevalence of illicit drug use per 100,000 population between 2005 and 2010 (National Survey on Drug Use and Substance Abuse – SAMHSA)	4.55	13.12

### Analytical Strategies

**Figure 2:**
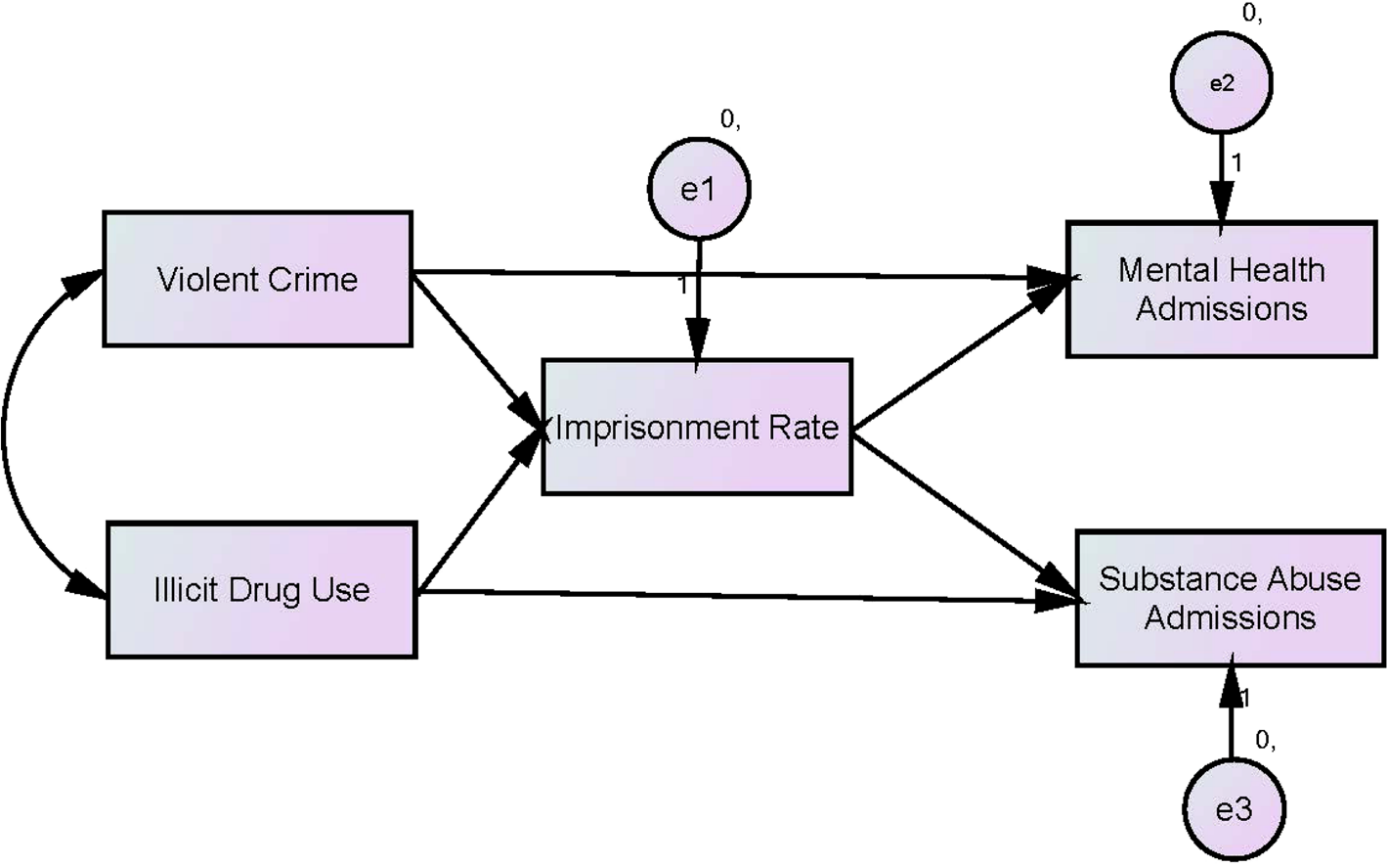
**The hydraulic model of institutionalization.**

The testing of the hypothesized inverse relationship between changes in the prison population and changes in the demands for community-based substance abuse and mental health services were executed in two stages. First, a bivariate analysis was performed to yield Pearson’s *r* coefficients to detect the shape, direction, and strength of the hypothesized associations. Then, a parsimonious path analysis model subjected the two hypotheses to a simple multivariate test. The path analysis model specified the flow of unidirectional impact from change in the prison population to changes in the substance abuse and mental health treatment populations in the community in the larger contexts of fluctuating levels of violent crime and illicit drug use (see Figure 2). Since both hypotheses were directional propositions asserting a negative relationship between changes in prison population and changes in community demands for substance abuse and mental health services, a one-tail significance test was implemented in both bivariate and path analysis models.

## Results

Table 2 displays the correlation matrix involving the five variables included in the analysis. Change in the state imprisonment rate was negatively correlated with change in the rate of substance abuse treatment admissions (r = −0.24; p < .05) and the change in the rate of inpatient admissions in state psychiatric hospitals (r = 0.10; p > .05) as predicted. Only the bivariate association between imprisonment and substance abuse treatment admissions attained the conventional threshold of statistical significance. It became important to see whether the direction and strength of these two moderate links between change in the prison population and the change in the substance abuse and mental health treatment populations in the community can withstand the multivariate test from the path analysis model.

**Table 2:** **Correlation matrix (N = 50 states)**

	**2**	**3**	**4**	**5**
1. Imprisonment Rate	−0.24*	−0.10	0.12	−0.17
2. Substance Abuse Admissions	-----	0.00	−0.05	−0.02
3. Mental Health Admissions		-----	−0.01	−0.20*
4. Violent Crime			-----	−0.02
5. Illicit Drug Use				-----

*p < .05 (one-tail test).

### Path Analysis Model

**Figure 3:**
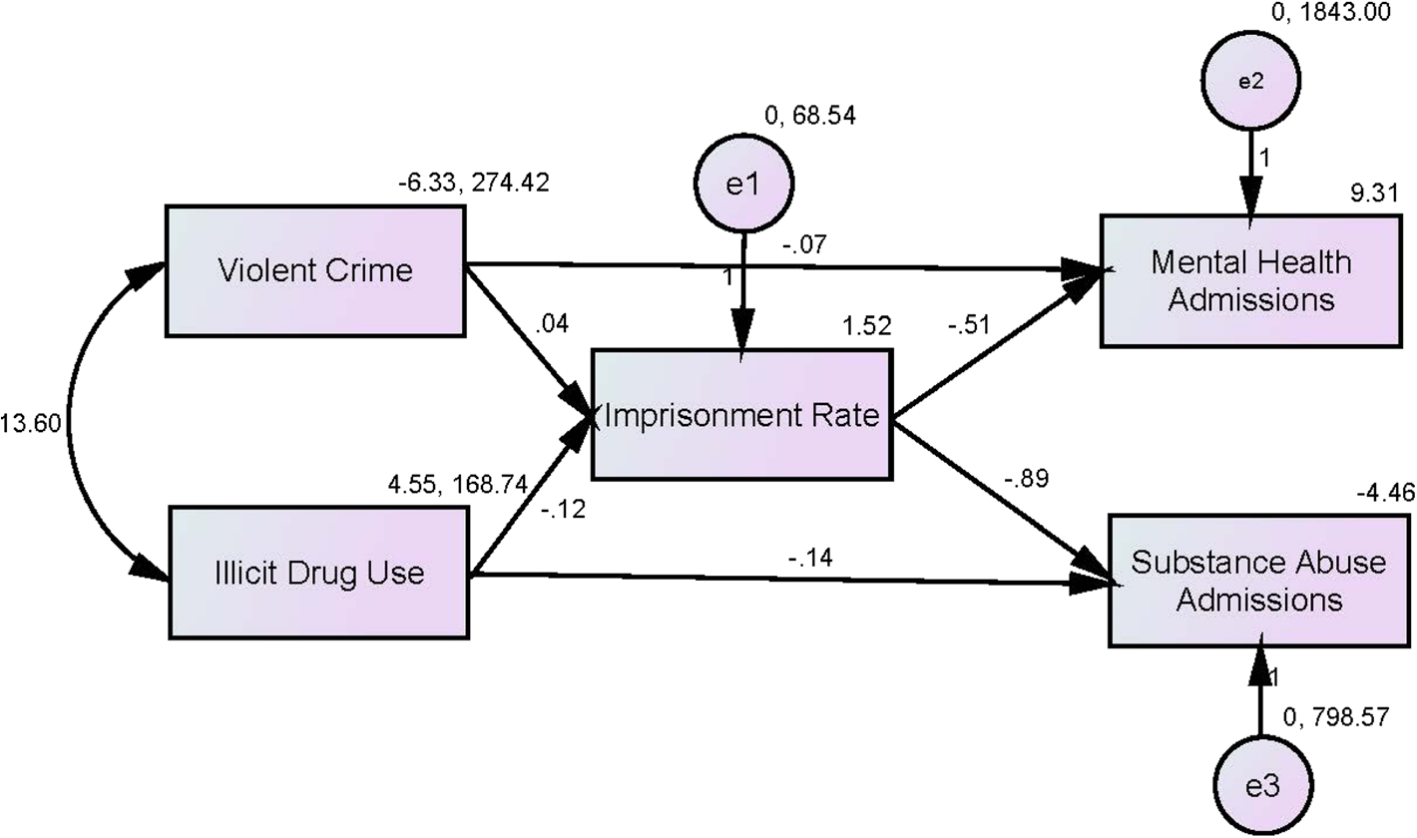
**Path analysis of the hydraulic model.**

The model Chi-square of 3.087 failed to attain the statistical significance level of .05 (see Table 3), which indicates that the fit between our reduced model and the data is not significantly worse than the fit between the just-identified model in which there would be a direct path from each variable to each other variable (see Figure 3). With a Normal Fit Index (NFI) of 0.652 (smaller than 0.9) and a Root Mean Square Error of Approximation (RMSEA) of 0.024 (smaller than 0.05), we conclude that the overall goodness of fit of the model is moderate but adequate (Kline 2010).

**Table 3:** **Model fit and effect coefficients of the path model (N = 50 states)**

Model fit			
Chi-square (sig. level)		3.087 (0.378)	
Norm Fit Index (NFI)		0.652	
Root Mean Square Error of Approximation (RMSEA)		0.024	
Effect coefficients of imprisonment rate			
	Unstandardized coefficient	Standardized coefficient	S. E.
Substance Abuse Admissions	−0.891*	−0.257*	0.035
Mental Health Admissions	−0.509	−1.000	0.243

*p < .05 (one-tail test).

Essentially, outcomes from the path analysis modeling replicated and corroborated the bivariate findings. Holding constant the direct and indirect effects of changes in the rates of violent crime and illicit drug use, change in prison population was negatively associated with changes in the rate of substance abuse (unstandardized coefficient = −0.891; p < 0.05) and mental health admissions (unstandardized coefficient = −0.509; p > 0.05) in the community. For every one percentage-point decrease in the imprisonment rate there was a statistically significant increase of a 0.89 percentage-point in the rate of substance abuse treatment admissions in the community. Likewise, every one percentage-point decrease in the rate of imprisonment was statistically non-significantly connected to a 0.51 percentage-point increase in the rate of inpatient admissions in state psychiatric institutions.

Both hypothesized associations were in the hypothesized direction, however, only one of them (i.e., the prison population-substance abuse treatment population link) was statistically significant. The relatively short window of observation (i.e., 2005–2010) may have restricted the range of within-case variation over time in this analysis of hydraulic transmission of institutionalized populations across systems. A longer time series data in the future may provide a better description of the phenomenon in the post-2000s decline in prison population. All in all, these outcomes provided partial but very encouraging support to the two research hypotheses.

### Further Research Implications

The concept of deinstitutionalization is longitudinal in nature; therefore, its measurement and analysis require that data and modeling techniques should be commensurate with this necessity. It should also be noted that while the term ‘deinstitutionalization’ may summarize a national trend, regional idiosyncrasies and patterns unique to individual states must be identified and interpreted. Two basic questions should guide the theoretical elaboration and methodological design of future epidemiological studies in prison deinstitutionalization: First, how does the process of deinstitutionalization evolve over time in each state? Second, what demographic, socio-economic, and political forces predict differences among states in their change? Other secondary questions will also have to be addressed in future research including: What is the shape of the mean trend in deinstitutionalization over time? Do past levels of incarceration predict the rate of change? Do two or more groups of states differ in their trajectories? How does rate of change or degree of curvature in the mean trend predict local crime rates and/or demands for mental health and substance abuse services? Does significant between-state variability exist in the shape of the trajectory?

On the policy side, the coincidence of the trend of prison deinstitutionalization with the implementation of the Affordable Care Act (ACA) of 2010, which is the most revolutionary piece of health care legislation in the United States in 45 years, calls for intensive monitoring from public health and criminal justice researchers. Together, these concurrent policy developments will have a lasting albeit still unknown impact on health outcomes for inmates, treatment of mental illness in the community, correctional health care costs and even recidivism. Two sets of ACA provisions should be of particular interest to researchers and policymakers. First, the federal government will help states expand Medicaid coverage by subsidizing 100% of expenditures for all individuals under age 65 with income below 133% of the federal poverty level who are not otherwise covered by Medicaid between 2014 and 2016. The sum of federal support is planned to decline gradually to 90% by 2020. Second, treatment of mental health problems and substance use disorders will be considered essential benefits. Accordingly, it is assumed that state governments may find powerful incentives in releasing non-violent inmates from jails and prisons while sentencing non-violent offenders to community-based sanctions so that the medical and psychiatric needs of these populations can be better attended in the community with federal funds. Future research will have to focus on the effects of the diversion of offenders with mental and substance abuse problems to the community, the potential increased investment on the continuity of care as a result of the reduced correctional health care expenditures, and the correlation between reductions in racial disparities in incarceration and more equal access to health care in the community. In addition, it is assumed that the landscape of services available for such at risk populations will evolve in such a manner that more somatic and mental health care needs will be addressed. Yet, these developments will also have to be examined in future studies.

## Discussion and policy implications

The physics principle of connected vessels determines that the amount of water in various connected vessels will always aspire to equilibrate. Such principle is based on the minimal potential energy principle suggesting that any system will remain in its lowest potential energy as long as the system enables smooth transition between different situations. Such principle also applies to the preliminary findings of this study; here we referred to it as the hydraulic transmission of institutionalized populations across systems. By using this principle we argue that social systems, similar to water moving in closed tubes, aspire to equilibrate; decrease in prison population will not go without a corresponding increase in community mental health and substance abuse services. Social voids like those created by deinstitutionalization must be filled; and with states deinstitutionalizing offenders the toll is on their corresponding communities to address the needs of those offenders who are reentering after being incarcerated. Yet, such demand for community services to the offenders’ population should not be taken lightly. As previously discussed in this article, some communities may require more services than others. Many incarcerated individuals are suffering from one medical condition or another, while others may be suffering from the morbidity of physical, mental, and substance/alcohol issues.

The prevalence of many chronic diseases, substance abuse, and mental health issues is much higher among incarcerated offenders compared to non-offenders (Dretsch 2013; Freudenberg 2001; Watson et al. 2004), and deinstitutionalization demands that such issues be addressed. This is more than just moving offenders from jails and prisons back to the community; this is about public safety and public health. We suspect that in the near future more incarcerated offenders will be released earlier to the community to alleviate conditions of overcrowding, and states’ budgetary crises. However, if we are to make this new deinstitutionalization movement work, proper planning and action must take place. Society still has responsibility for the safety and health of all its members, and that includes those released from our correctional facilities. With the recent approval of the Affordable Care Act (ACA) of 2010, released offenders will become eligible and, as intimated previously, have better access to health coverage in 2014. Consequently, it is assumed that such availability to health-related services in the community, to many individuals who were lacking it before the ACA, will likely reduce criminal behavior among mentally ill and those battling addiction. Yet, the ACA does not guarantee that all those who are in need for psychiatric care will follow their treatment plan. Some simply stop attending their treatment sessions, while others stop taking their medications because they think they do not need them anymore and can do without them. Such course of action will require better outreach and community monitoring to those in need.

As discussed by Lamb and Bachrach (2001), deinstitutionalization is not a one step process, but rather has three parallel phases: (1) the release of individuals from the institution into the community; (2) the diversion of individuals from entering the institution; and (3) the development of alternative community services. Consequently, any policy that wishes to be successful in the prison deinstitutionalization movement should involve considerable planning that will target all three phases, by properly acknowledging the alternatives to incarceration and institutionalization, and how such alternatives are better equipped to address the needs of those individuals targeted by the policy. We suggest, that more should be done at intake stages to identify the multiple and complex needs and risks of the convicted offender. This will enable criminal justice agents to tailor a more appropriate and individualized course of action, while also coordinating what services are needed and in what specific geographic locations. Partnering with agencies in the community is thus of paramount importance to the process, as such agencies will absorb and treat those who are convicted, as well as those who have already served time. As mentioned earlier in this section, the landscape of community based healthcare intervention is about to change over the coming years. As the U.S. grapples to unwrap the ACA and estimate the magnitude of its surprise, various challenges will, without doubt, affect criminal justice clients and the criminal justice system. Enrolling individuals involved in the criminal justice system into Medicaid, diverting large numbers of mentally ill and substance abusing offenders from criminal justice solutions to community-based healthcare interventions, and fostering partnerships will be one of the biggest challenges of both deinstitutionalization and the ACA; two mammoth challenges that share similar interests, and perhaps even similar destinies.

### Implications for Provision of Treatment Service Providers

In devising a policy and practice strategy to address the projected increase in the reentry population, leadership within communities for social and supportive services to ex-prisoners, specifically treatment services should be of primary concern. This is especially true in those communities with large counts and densities of returning ex-offenders, as they face even greater challenges of pro-social integration and improved public safety. The findings of this study support the need for organizations that provide treatment services to improve accessibility to ex-prisoners and improve pro-social reintegration for ex-offenders in their communities. Specifically, reentry stakeholders should be encouraged to take a leadership role in building the outreach and networking capacity for treatment services for ex-offenders. This step, which consists of outreach, networking, community justice/reentry partnerships and funding resource building, requires strategic planning, implementation, and evaluation. These organizations should develop policies which would: (1) engage in more outreach to ex-prisoners; (2) engage in more networking between other service providers, community organizations and community justice and governmental agencies; and (3) seek more reentry funding to increase provision of treatment services.

### Outreach

Several elements of outreach are relevant at this juncture. First, ex-prisoners need to be made aware of mental health services prior to release. Most often ex-offenders do not get connected to the treatment services they need. Accordingly, it is vital to examine the experiences and preparedness of those individuals revolving through the criminal justice system, and how the system addresses their special needs, be they mental health, substance abuse, or any other health related needs (Gideon 2013). These are essential for orchestrating smoother transitions from incarceration back to the community. Such practice is known as *discharge planning*, and it provides the critical link between prison-based intervention process and the transition back to the community. The ultimate goal of the discharge plan is to link inmates with appropriate health and other human service providers in the community (Mellow et al. 2008), in order to ease the transition and to reduce the risk of recidivism. An essential part of the discharge plan is the need for outreach programs that will identify and direct needed individuals in the right direction. Specifically, the process of outreach must begin during pre-release and continue through the last stage of reentry, called post-supervision. Research has found that only about a third of organizations engage in pre-release outreach (Frazier 2008).

Mental health organizations should be encouraged and provided opportunities by correctional facilities at every level (federal, state, and county) to do the same. Secondly, outreach must address issues of eligibility and accessibility. Individuals returning from prison and jail face an array of challenges: not only are they grappling with feelings of inadequacy, low self-esteem and hopelessness, they must also negotiate the bureaucratic hurdles required by many agencies. In a study by Frazier (2008), providers stated that making the requirements and processes clear and intelligible is an initial step that could assist ex-prisoners. Thirdly, community organizations of all types should be encouraged to share information on services available to ex-prisoners, especially those that have completed their sentences and are no longer connected to the criminal justice system. A concentrated effort must be made throughout the entire community to create more awareness of services that organizations claim they are able to provide, especially services that assist in successful reintegration. These organizations, as well as ex-prisoners, should also be engaged in finding new and better ways to reach out to individuals returning from prison or jail, connecting them with services.

### Networking

Taking the leadership role of networking should be addressed on two fronts: community justice/reentry partnerships, as well as both formal and informal networking among all organizations within the community, even those that do not provide social and supportive services to ex-prisoners. Community justice/reentry partnerships take place between criminal justice agencies (i.e. the courts, parole and probation, and the police) and community organizations and institutions. Presently, few organizations collaborate with parole and probation agencies at the federal, state, or county levels (Frazier 2008). These and other collaborations should be encouraged to grow and continue, while new ones should be encouraged to develop. The benefits of such networks considerably improve the provision of services and other desired outcomes, such as improved outreach.

### Funding

Because funding is the primary barrier to increased provision of treatment services, leadership is needed to promote education and awareness of funding streams and processes. Since many treatment service organizations lack the resources necessary to seek and successfully apply for additional potential funding, leadership in partnering with funding sources to provide workshops and seminars that could be valuable for organizations seeking to serve more ex-prisoners. Leadership is also needed to determine and share best practices that may improve capacity without increased funding. In other words, more direction on ways to improve efficiency and effectiveness would also help organizations to improve their ability to serve more ex-prisoners.

## Conclusion

Deinstitutionalization should not be about becoming more lenient or less punitive; it should be about being pro-social and supportive of the reintegration of ex-offenders while maintaining the health and safety of the community. Years of valuable research informs us that we cannot, and should not, tolerate practices of locking up people as the sole means of punishment (Clear 1994), as such practice have a negative effect on our communities. Ignoring the needs of the incarcerated as well as those of society, does more harm than good, and will result in the failure of deinstitutionalization as once before experienced by the deinstitutionalization of the mentally ill. Further, failure to address the needs of those diverted, and otherwise released from prison may also result in increase of violent crimes, which in turn, will create a new cry for more incarceration. Such destructive cycle must be broken if the present prison deinstitutionalization movement is not to mirror that of the previous mental health deinstitutionalization movement.

While the present study focuses on mental health and substance abuse treatment, every area of healthcare (HIV/AIDS and primary health care, alcohol and substance abuse treatment, and mental and behavioral health treatment) is seen to be of great need within the community. In addition, in-patient treatment is among the least provided services and these providers have the least capacity to serve more ex-prisoners (Frazier 2008). Effort and great attention must be given to each individual healthcare arena instead of viewing them in concert. The various services, issues, and treatments must be viewed separately and we must remember that each provider type may face somewhat different challenges. Accessibility not only includes issues of location and proximity to service providers, it also includes access to treatment and available treatment options.

Additionally, mental illness and substance abuse—often co-existing disorders—place ex-prisoners at high risk of re-incarceration, reconviction, or re-arrest and also contribute to instability in employment and housing. Some ex-prisoners are treated for both mental illness and substance abuse while incarcerated; however, once released, most often there is no continuum of care. Even fewer ex-prisoners receive treatment for HIV/AIDS because there is no mandatory testing for inmates (Macalino et al. 2004; Drucker 2011; Maruschak et al. 2012). While strict Health Insurance Portability and Accountability Act of 1996 (HIPPA) laws prevent sharing of patient information, efforts must be made to improve the continuum of care for those receiving treatment while incarcerated. Attention must also be given to building partnerships to share referrals and find other ways to provide these desperately needed healthcare services. With the ACA the potential barriers to the treatment of those offenders diverted/deinstitutionalized should be identified and resolved before it is too late. Positioning available services to released offenders, and those who are in the community and foster such needed partnerships will determine the success of both movements.

### Study Limitations

Readers should be aware of two limitations in our data, one of theoretical nature and the other with methodological implications. First, the use of change in state imprisonment rates as the mediating variable in the model did not allow the discrimination of prison admissions from prison releases. While most discussions have focused on penal policy reforms leading to decreases in prison populations, it is also true that violent crime rates have dramatically declined across the country since the 1990s. Therefore, recent reductions in prison populations could have been triggered by both decreased admissions (due to crime decline and a more widely use of diversion programs) and increased releases. Since the hydraulic model hypothesized in this analysis assumed a relative stability in the total size of institutionalized populations and an inverse correlation between correctional institutionalization and psychiatric institutionalization, the exclusion of measures of prison admission and release did not affect the goodness of data for the testing of the two hypotheses.

Second, time series data used in this analysis pertained to the 2005–2010 period. This relatively short span of observation rendered a longitudinal sample with a limited number of years, which may have restricted the statistical power of our analysis. As a result, the risk of type II error or the failure to reject a false null hypothesis was high, making our results conservative in nature. That is, the probability of missing the true effect of prison populations on community treatment populations was much higher than the probability of detecting the effect when it did not exist.
